# Analysis of health promotion and prevention financing mechanisms in Thailand

**DOI:** 10.1093/heapro/daw010

**Published:** 2016-03-17

**Authors:** Akihito Watabe, Weranuch Wongwatanakul, Thaksaphon Thamarangsi, Phusit Prakongsai, Motoyuki Yuasa

**Affiliations:** 1 Department of Public Health, Graduate School of Medicine, Juntendo University, Japan; 2 Department of Health System Governance and Financing, World Health Organization, Geneva, Switzerland; 3 International Health Policy Program, Nonthaburi, Thailand; 4 Department of Non-Communicable Diseases and Environmental Health, South East Asia Regional Office, World Health Organization, New Delhi, India

**Keywords:** economic analysis, population health, sustainable development, evaluating health promotion, health policy analysis

## Abstract

In the transition to the post-2015 agenda, many countries are striving towards universal health coverage (UHC). Achieving this, governments need to shift from curative care to promotion and prevention services. This research analyses Thailand's financing system for health promotion and prevention, and assesses policy options for health financing reforms. The study employed a mixed-methods approach and integrates multiple sources of evidence, including scientific and grey literature, expenditure data, and semi-structured interviews with key stakeholders in Thailand. The analysis was underpinned by the use of a well-known health financing framework. In Thailand, three agencies plus local governments share major funding roles for health promotion and prevention services: the Ministry of Public Health (MOPH), the National Health Security Office, the Thai Health Promotion Foundation and Tambon Health Insurance Funds. The total expenditure on prevention and public health in 2010 was 10.8% of the total health expenditure, greater than many middle-income countries that average 7.0–9.2%. MOPH was the largest contributor at 32.9%, the Universal Coverage scheme was the second at 23.1%, followed by the local governments and ThaiHealth at 22.8 and 7.3%, respectively. Thailand's health financing system for promotion and prevention is strategic and innovative due to the three complementary mechanisms in operation. There are several methodological limitations to determine the adequate level of spending. The health financing reforms in Thailand could usefully inform policymakers on ways to increase spending on promotion and prevention. Further comparative policy research is needed to generate evidence to support efforts towards UHC.

## BACKGROUND

The 2030 Agenda for Sustainable Development emphasizes the importance of achieving universal health coverage (UHC) in reaching its third goal, to ensure healthy lives and promote well-being for all at all ages (United Nations, 2015). In pursuing this goal, many low- and middle-income countries harbour strong aspirations to make everyone access to essential health services including health promotion, prevention, treatment and rehabilitation, without suffering financial hardships (WHO, 2010), and are considering their health financing reforms for that. However, the majority of discussions on health financing reforms continue to focus heavily on curative care, leaving health promotion and prevention out of scope.

Tangcharoensathien *et al.* (Tangcharoensathien *et al.*, 2008) estimated that across 120 countries in 1999–2003, only 2.9% of the total health expenditure (THE) was spent on health promotion and prevention. As the global burden of diseases shifts from communicable to non-communicable diseases (NCDs) (Murray *et al.*, 2012), essential health services will need to shift increasingly towards health promotion and prevention (Boerma *et al.*, 2014). Without this, countries will struggle to ensure healthy lives and well-being of their populations, and will likely face serious escalation of healthcare costs. Financial sustainability and security for health promotion and prevention, therefore, are important and should be integrated more comprehensively into national financing strategies towards achieving UHC.

Over the past two decades, a number of innovative financing schemes to address health promotion have been tested. An example of this, from health system reforms in Mexico, was the separation of funding agencies for personal health services and public health services (Frenk *et al.*, 2009). The Fund for Community Health Services was created to finance public health services which included health promotion, immunization and the control of diseases. In some countries, Health Promotion Foundations were established to overcome chronic financial constraints for health promotion (WHO, 2007). Schang *et al.* (Schang *et al.*, 2012) reviewed Health Promotion Foundations in five high-income countries and suggested that it could be an alternative model for securing funds for health promotion. Vathesatogkit *et al.* (Vathesatogkit *et al.*, 2013) also introduced 18 case studies of innovative health promotion financing schemes across 14 countries and 4 states, which included Thailand. However, these studies did not make clear how the schemes sit within the overall health financing systems (Nam and Engelhardt, 2007; Prakongsai *et al.*, 2007; Bayarsaikhan, 2008).

Thailand established two independent public funds for health during their health financing reforms (Prakongsai, 2007). There have been some reviews related to health financing and health promotion in Thailand (Adulyanon, 2012; Evans *et al.*, 2012); however, they only focus on one fund or on curative care. None of the studies depict the entire picture for financing health promotion and prevention in Thailand, nor is it clear how and how much the innovative financing scheme contributes to UHC. This paper, therefore, analyses the complete structure and function of Thailand's financing system for health promotion and prevention, and critically assesses policy options in the context of health financing reforms towards achieving UHC.

## METHODOLOGY

In the context of UHC, health promotion and prevention can be divided into two approaches: service based and population wide (Frenk *et al.*, 2009). In this paper, service-based approaches will be defined as promotion or prevention services, which are provided to individuals through healthcare or public health providers. This includes services such as mammograms, pap smears, antenatal care and measles vaccinations (Boerma *et al.*, 2014). Population-wide approaches will refer to services and activities that target a large group of people, such as improving a water source, ensuring adequate sanitation and promoting the non-use of tobacco (Boerma *et al.*, 2014).

The study combines a number of methodologies and multiple sources of evidence, including scientific and grey literature, quantitative data from the National Health Accounts, and qualitative interview data from key stakeholders in Thailand. Data were collected during June and July 2013.

Scientific and grey literatures were reviewed to analyse the current structure and function of promotion and prevention financing in Thailand using a well-recognized health financing analytical framework (Kutzin, 2001). The literature included relevant legal texts, policy documents and external review reports in English and Thai.

Original expenditure data between 1994 and 2010 collected for the National Health Accounts by International Health Policy Program, Thailand, were used to estimate the proportion of promotion and prevention expenditure as part of THE in Thailand. We assumed that health care function six (HC.6) of the OECD System of Health Accounts version 1.0 would be the total expenditure on prevention and public health (TEPP) (OECD, 2000; Poullier *et al.*, 2002; Tangcharoensathien *et al.*, 2008). HC.6 includes HC.6.1 maternal and child health, family planning and counselling; HC.6.2 school health services; GC.6.3 prevention of communicable diseases; HC.6.4 prevention of NCDs; UC.6.5 occupational health care; and HC.6.9 all other miscellaneous public health services. According to the World Bank's income group classification (World Bank, 2011), Thailand has moved from a lower-middle-income country to an upper-middle-income country in 2011, which means that Thailand was in transition from one to another group during the time frame of this study. Therefore, as reference points with which to compare, promotion and prevention expenditures among lower-middle and upper-middle-income countries in 2005–11 were estimated using WHO Global Health Expenditure Database, TEPP as a proportion of THE among 29 and 28 countries, respectively. All fiscal data are reported in current US dollars in 2011 and current Thai baht.

Interviews with key stakeholders involved in Thailand's health financing reforms were conducted to supplement preliminary findings based on the review of literature and health expenditure analysis, as described above. In July 2013, 12 face-to-face interviews were conducted using a semi-structured in-depth interview guide, which provided a framework while still allowing enough flexibility to collect unsolicited information (Gray, 2009). Key interviewees were selected from executive officers of public agencies, heads of departments in government and health providers.

Ethical approval was gained from the Ministry of Public Health (MOPH) Research Ethics Committee in Thailand (reference number: 1171/2556). There are no conflicts of interest to be declared.

## RESULTS

### Thai health financing system for health promotion and prevention

**Fig. 1:**
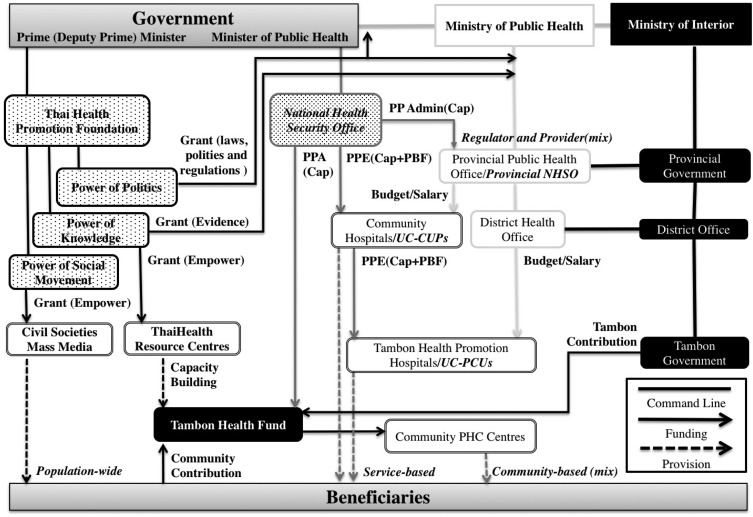
Promotion and prevention financing and service stakeholders in Thai Health Systems.

Source: Diagram created by the authors based on Thailand Health Profile 2008–10, MOPH, Nonthaburi and interviews.

Before 2001, the MOPH in Thailand was the main provider of health promotion and prevention services. In 2002, Thailand declared it had achieved UHC, after the newly elected government introduced the Universal Coverage (UC) scheme managed by the National Health Security Office (NHSO) to fill the population gap not previously covered by existing health schemes limited only for civil servants, the Civil Servant Medical Benefit Scheme (CSMBS) and for formal workers, the Social Security Scheme (SSS) (Hanvoravongchai, 2013). Unlike CSMBS and SSS, the UC scheme covers both preventive and curative care. In 2001, the Thai Health Promotion Foundation (ThaiHealth) was also established in accordance with the Thai Health Promotion Foundation Act 2001. It was designed to empower civil society and promote the well-being of citizens, by providing financial support for projects that change social values, lifestyles and environments conducive to improved health (Siwaraksa, 2011). By 2013, there were three agencies plus local governments who share major funding roles for promotion and prevention services in Thailand. Key features and interactions between these funds, organized by the predominant pooling body, are summarized based on interviews in Figure 1. Table 1 provides further details in revenue, pooling, purchasing and service provision of the funds.

**Table 1:** Comparison of three key financing schemes for promotion and prevention in Thailand

	Thai Health Promotion Foundation	NHSO UC—Promotion and Prevention		MOPH
		PPE	PPA	
Prevention approach	Population wide	Service based	Community based (mix)	Regulator and provider (mix)
Pooling body	Independent public fund	Independent public fund		Government body
Governance	Prime (Deputy Prime) Minister	Minister of Public Health		Minister of Public Health
Legislation	Health Promotion Act	National Health Security Act		National Health Act
Revenue source	2% Surcharges of alcohol and tobacco taxes	General taxes		General taxes
Allocation method	Earmarking	Per capita/10–15% fixed allocation		Line-item budget
Fiscal cycle	Project base (1 or 6 months/1 or 3 years)	Annual		Annual
2010 Annual budget million US$ (per capita US$)	Project grants: 128	Prevention Service Package: 470 (7.2), PPE: 248 (3.8), PPA: 118 (1.8)		Programme budget: 308
Purchasing mechanism	Proactive and flexible grants	Capitation (75%), PBF (25%)	Capitation	Programme budget
Provider	Policymakers, researchers, mass media, civil society	Healthcare providers	Community volunteers	Healthcare providers, public health providers

Source: Table created by the authors based on Thailand Health Profile 2008–10, MOPH, Nonthaburi; UC scheme guideline 2013, NHSO, Nonthaburi; and 10 years review of ThaiHealth, ThaiHealth and interviews.

Thai Health Promotion Foundation (ThaiHealth) was designed to finance population-wide promotion and prevention activities. The law regulates revenue for ThaiHealth to be directly transferred from a 2% surcharge on tobacco and alcohol taxes and pooled in an independent public fund governed by the Prime (Deputy Prime) Minister. The fiscal cycle of ThaiHealth project grants vary from 1 month to 3 years. ThaiHealth's budget trend shows a remarkable increase to secure the population-wide activities since it was launched in 2011, from 47 million US$ in 2001 to 128 million US$ in 2011 (Galbally *et al.*, 2012), yet some interviewees complain that this scheme is problematic in terms of transparency. They argue that ThaiHealth receives amount of budget without requiring annual negotiation with the Ministry of Finance and approval by the parliament. Another interviewee mentions that ThaiHealth has experienced continuous pressures from politicians to influence this fund for their political tools. Addressing these challenges, this fund is regulated by law and strict internal policies in conflicts of interests (Carroll *et al.*, 2007). ThaiHealth adopts three approaches to leverage its fund; power of knowledge where activities create evidence in health promotion; power of politics where activities develop laws, policies and regulations in health promotion; and power of social movements where activities empower civil societies to promote health (Carroll *et al.*, 2007). Interviewees acknowledged that ThaiHealth acts as catalysis to accelerate innovative ideas with proactive and flexible grants and diverse network with government, public and private sectors and civil societies, which contributes multi-sectoral approach in health promotion and prevention. In the power of knowledge approach, ThaiHealth funds a number of semi-autonomous institutes under MOPH, which produce evidence around health policies and economics. As for power of politics, ThaiHealth funds government committees to accelerate their policy development process, such as the national health assembly, an alternative mechanism to discuss and recommend health issues directly from civil societies to the cabinet. In the area of social movement, ThaiHealth provides grants to civil societies and mass media, such as the Stop Drink Network, which connects around 1000 local leaders and 300 NGOs for alcohol control programmes. In addition to the grant scheme, ThaiHealth owns 30 resource centres that assist civil societies in applying, using and accounting for its funds efficiently and appropriately.

NHSO is an independent public fund governed by the Minister of Health and purchases service-based preventive and curative care. Every year, a lump sum per capita is allocated to prevention and promotion services (UC-PP) (NHSO, 2013a). An interviewee mentions that NHSO faces more difficult to convince the government in order to secure the capitation for preventive services due to less robust evidence than curative services. Therefore, the proportion of UC-PP has been marginalised from 15 to 10% of the UC budget by a higher increase in curative care. In 2013, 470 million US$ (7.20 US$ per capita) was allocated from government general taxes to these services for the entire population (65.4 million) (NHSO, 2013b). Under the prevention and promotion express-based payment (PPE) system, 248 million US$ (3.8 US$ per capita) was used for contracting units for primary care (CUPs) and primary care units (PCUs) provide service-based prevention (Evans *et al.*, 2012). In 2013, NHSO also introduced performance-based financing (PBF) for 18 services (NHSO, 2013b). Seventy-five per cent of PPE is paid prospectively through age risk-adjusted capitation, while the remaining 25% is paid retrospectively if providers have achieved annual performance-based targets set by NHSO in consultation with MOPH, for instance, 45% of the population above 15 years of age receive metabolic screening or 13% of females between 30 and 60 years old receive pap smear screening in 2010–13 (NHSO, 2013b). A director of a CUP is responsible for the overall financial and outcomes management of the CUP as well as some PCUs in the assigned area. Approximately 80% of CUPs and PCUs are public facilities. Through Tambon Health Insurance Funds (THIFs), prevention and promotion area-base payment (PPA), amounting to 118 million US$ (1.8 US$ per capita), supports primary health care (PHC) centres to implement their community-based prevention activities. Some interviewees suggest that ThaiHealth leverages its approach with the UC-PP scheme as THIFs fund some best practices initially invested by ThaiHealth. Such a relationship between NHSO, THIFs and ThaiHealth enhances synergy effects. In addition, an interviewee states that resource allocation to specific areas and hospitals used to be unclear due to political pressure; on the other hand, the current NHSO resource allocation formula is clear and fair to all health providers and facilities with less political interference. Unlike MOPH, the health promotion budget under UC scheme is set by capitation and performance-based payment; therefore, it is expected that there is less room to manipulate resource allocation.

The primary role of the MOPH is to develop health policies, enforce regulations and provide health services at the national, provincial, district and tambon levels (MOPH, 2011b). Despite some interviewees accept that MOPH is constrained funding capacity by line-item budget, MOPH remains one of the major actors as the regulator and providers of both approaches (MOPH, 2011a). Their funding is allocated through a line-item budget from general taxes and covers activities related to the National Priority Programs. This research estimates that the total budget for health promotion and prevention was ∼308 million US$ in 2010, based on three programmes that are tightly related to health promotion, namely disease prevention/control and health promotion, health system development and drug abuse prevention and resolution (MOPH, 2011b). Although there was a critical tension before, some interviewees said that the relationship between MOPH and NHSO is moderate these days. The other interviewees are even concerned that NHSO is getting too close to MOPH, which would spoil the advantages of the purchaser–provider split. For example, a chief of a Provincial Public Health Office, the local health authority under the MOPH, doubles a head of a provincial NHSO. MOPH is able to exert influence by regulating, licensing and adjusting health workforce. On the other hand, the relationship between MOPH and ThaiHealth is rather problematic. MOPH used to control most of the health prevention and promotion interventions; thus, MOPH seems frustrated with the limited influence to this fund.

Thailand is divided into 77 provinces, and each province is divided into districts, which are further divided into sub-districts, called *tambon*. THIF is the tambons' pooling body for community health, supervised by a Province Governor, under the Ministry of Interior (MOI) (Orajit, 2010). In addition to PPA from NHSO, THIF is co-funded by the tambon government, with contributions ranging from 10 to 50%, depending on its capacity. Community health volunteers in community PHC centres provide community-based health activities (mixed approaches). An interviewee mentioned that some THIFs also receive financial or technical supports from ThaiHealth to implement community-based activities. The activities funded by THIF vary in each tambon, based on its community's priority.

### Prevention expenditure trend

**Fig. 2:**
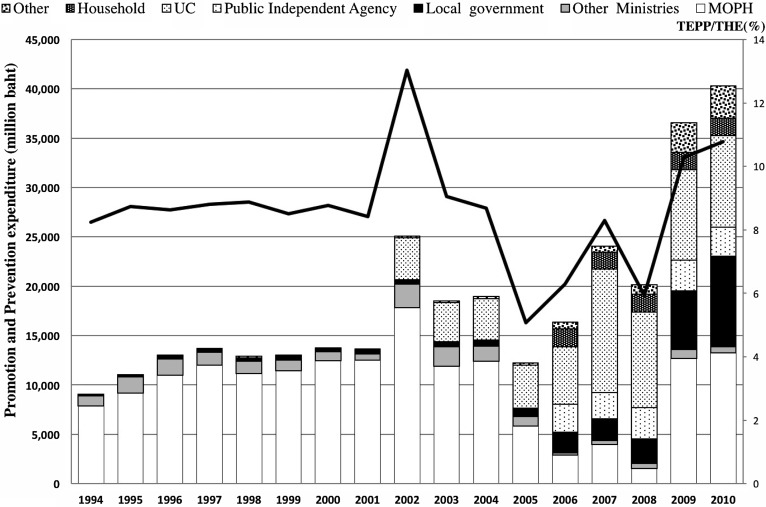
The trend of promotion and prevention expenditure in Thailand (1994–2010).

Source: Chart created by the authors based on National Health Account data set in Thailand (1994–2010), International Health Policy Program, Nonthaburi.

In Thailand, THE was 3.9% of gross domestic product (GDP) in 2010, which was below 7.0% of the GDP spent on average by upper-middle-income countries in 2010. However, compared with other countries with a similar level of national resources, government expenditure on health is high (75.8% of THE and 14% of general government spending) with low private health expenditure (24.2% of THE) and very small external sources (0.1–0.3% of THE) (Tangcharoensathien *et al.*, 2010). According to the National Health Accounts of Thailand, TEPP constituted 8.3% of THE in 1994, growing to 10.8% in 2010 (Figure 2). In total, 10.8% of THE is above the average for both lower-middle- and upper-middle-income countries, who spent ∼9.2 and 7.0% of THE, respectively. In 2010, the share of MOPH expenditure as a proportion of TEPP was 32.9%, followed by the UC scheme and local governments at 23.1 and 22.8%, respectively. The expenditure of the Public Independent Agency, serving as a proxy for ThaiHealth, accounted for 7.3% of TEPP. It was also noted that household payments contributed 4.4% of TEPP. Other ministries only contributed 1.5% of TEPP and 8% aggregated from all the other sources. Although the 10-year average TEPP as the percentage of THE was 8.6%, it has fluctuated over the past decade for several reasons. Prior to 2002, MOPH expenditure was the predominant form of promotion and prevention funding in Thailand, though this was hiked up in 2002, then dropped between 2005 and 2008, but after 2009 it recovered at the same level. Multiple interviewees pointed out that the political instability and government changes affected the expenditure trends in 2002 and during 2005–8 due to budget terminations and carry-overs as well as changes in expenditure data inclusion criteria. In addition, expenditure data for ThaiHealth were only available since 2005.

## DISCUSSION

Countries that seek to introduce health financing reforms towards UHC need to find adequate and sustainable revenue sources for health promotion and prevention if the 2030 Agenda for Sustainable Development is to be realized. Thailand's health financing system, for promotion and prevention in particular, is strategic and innovative, involving three different mechanisms plus local governments which complement each other. Government line-item budgets, such as those employed by the MOPH, are usually rigid and not easy to increase, especially for new challenges such as NCDs. However, the two independent public funds financed by earmarked taxes and capitation-based allocations are able to provide additional revenue for such needs. In addition to the support from two central funds, local funds also mobilize community resources for prevention.

While political instability disrupted implementation of some prevention and promotion activities during 2004–8, the two public funds remain independent without undermining the role of the MOPH. In addition, it should be noted that the expenditure of ThaiHealth from earmarked budget is relatively small (7.3%) compared with the ones of NHSO (23.1%) from capitation-based budget and MOPH (40%) from line-item budget. By splitting the roles of providers and purchasers, ThaiHealth and NHSO are able to employ tools for strategic purchasing to select service providers more flexibly and let them provide targeted prevention services more efficiently. The complementary relationship of ThaiHealth and NHSO also enhances synergistic effects on prevention services. NHSO prioritizes equity in access to service-based approaches for all, while ThaiHealth focuses on high-performance investments for population-wide approaches. The role of ThaiHealth is rather catalytic and leverages innovative ideas with flexible funding to a wide range of multi-sectoral networks. Furthermore, the share of local government is increasing (22.8%) in order to address different communities' needs for promotion and prevention. More efficient use of available resources is equally important in health financing reforms by assigning appropriate role of providers and purchasers.

The reforms in Thailand could be an interesting example for policymakers looking to increase their share of spending on TEPP; however, other countries may face several challenges to adopt this policy option. First of all, the Ministry of Finance, politicians and many economists disagree with dedicated taxes due to inflexible and uncontrollable revenue (Carol, 2004). Secondly, strong political leadership will be crucial to convince stakeholders especially if some budget and functions of the Ministry of Health are transferred to the new agencies (Siwaraksa, 2011). Thirdly, policymakers and politicians do not usually acknowledge the importance of preventive care and activities, and tend to prioritize access to curative care to satisfy beneficiaries (Prakongsai *et al.*, 2007). Finally, rapid economic growth and/or political change might be a prerequisite for such a drastic system reform (Tangcharoensathien *et al.*, 2011; Paper *et al.*, 2012).

Alternatives options would be one independent public fund financed by capitation-based and fixed proportion of general budget. Similar to the UC scheme in Thailand, predictable revenue sources could ensure both individual service-based prevention and some of population-wide health promotion activities. The independent public fund from the health ministry enables the fund to adopt innovative approach without facing bureaucratic hurdles (Vathesatogkit *et al.*, 2013) and can beyond its scope outside the health sector to address social determinant factors in health (Leppo *et al.*, 2013). Moreover, separating purchasers from service providers would contribute efficiency of health services (Evans *et al.*, 2012). Law or strict internal regulation could help the fund to stay away from corruption. Strategic purchasing, such as capitation combined with PBF, can be a good option to consider in other countries (Kalk *et al.*, 2010). An advantage of single fund is to save extra administration costs for pooling, purchasing and coordination (Gottret and Schieber, 2006). In order to prevent unnecessary fragmentation and complication, good coordination mechanisms and fewer pooling bodies are crucial. However, a potential downside of single pooling is that health prevention and promotion have a risk to be marginalized by curative care as seen in the case study of the UC scheme. Therefore, careful design of legislation or regulation is critical to secure a set amount of financings to both service-based and population-wide health promotion and prevention (WHO, 2007).

This research has a number of limitations that need to be considered. It is a challenge to determine the target level of expenditure for promotion and prevention services. This research adopts an indicator, TEPP as the percentage of THE, to measure the adequacy and sustainability of prevention expenditure. The Abuja Declaration (WHO, 2001) recommends that governments invest more than 15% of total government expenditure in health, and another oft-cited expenditure target to ensure universal PHC services in low- and middle-income countries is 86 US$ per capita (Mcintyre and Meheus, 2014). Yet, there is no international consensus on what is deemed appropriate expenditure for promotive, preventative, curative or rehabilitative approaches. Furthermore, the inclusion criteria of services included in health care function six were not clear, in particular population-wide approaches; therefore, some expenditure might have been excluded from our analysis. This includes expenditure from ThaiHealth, which was not in health care function six between 2001 and 2005. As coding practices for expenditure broaden (OECD *et al.*, 2011), further research will be needed to assess whether indicators could exist that measure adequacy and sustainability of promotion and prevention services. Finally, this research includes all relevant and available local articles in English and conducted interviews, yet only four original articles written in Thai were used for thematic analysis. The interview was balanced and included major stakeholders based on neutral expert recommendations; however, a limited number and background of interviewees might have excluded diverse opinions. More comparative policy research on financing health promotion and prevention is required to better inform future policy reforms in all counties.

## CONCLUSION

Towards achieving UHC in the post-2015 era, governments will need to find additional domestic resources and develop strategies to shift their focus towards health promotion and prevention. The primary objective of this research is to analyse overview of Thailand's financing system for health promotion and prevention and critically assess whether this model can be applicable to other countries in the context of health financing reforms towards achieving UHC. The financing scheme for health promotion and prevention in Thailand is unique as two independent public funds financed by different source of revenue and for different prevention purpose co-exist with the health and local authorities: ThaiHealth for a population-wide approach financed by earmarked taxes, the UC-PP scheme mainly for service-based approach financed by capitation-based and fixed allocation from general UC budget. The reforms in Thailand, as documented in this paper, could be an interesting example for policymakers looking to increase their share of spending for prevention as well as to efficiently use available resources for it. However, other countries may face challenges to adopt this policy option politically. This research suggests an alternative option, an independent public fund financed by capitation-based and fixed proportion of general budget and financing both approaches. Although there is no one size fit model and policy decision must be made based on a specific country's context, achieving and further sustaining UHC, it is crucial to make sure that health promotion and prevention are included in the essential benefit package. Thus, more comparative policy research is required to generate the much-needed evidence on financing health promotion and prevention.
